# Untargeted Metabolomics Analysis Using UHPLC-Q-TOF/MS Reveals Metabolic Changes Associated with Hypertension in Children

**DOI:** 10.3390/nu15040836

**Published:** 2023-02-06

**Authors:** Kexin Zhang, Yanyan Liu, Lingyun Liu, Baoling Bai, Lin Shi, Qin Zhang

**Affiliations:** 1 Beijing Municipal Key Laboratory of Child Development and Nutriomics, Capital Institute of Pediatrics, Beijing 100020, China; 2 Department of Cardiology, Children’s Hospital Affiliated to Capital Institute of Pediatrics, Beijing 100020, China

**Keywords:** hypertension, overweight, hyperhomocysteinemia, metabonomics, children

## Abstract

The mechanism of hypertension in children remains elusive. The objective of this study was to analyze plasma metabolomics characteristics to explore the potential mechanism of hypertension in children. Serum samples from 29 control children, 38 children with normal body mass index and simple hypertension (NBp), 8 children overweight with simple hypertension (OBp), 37 children with normal body mass index and H-type hypertension (NH) and 19 children overweight with H-type hypertension (OH) were analyzed by non-targeted metabolomics. A total of 1235 differential metabolites were identified between children with hypertension and normal controls, of which 193 metabolites including various lipids were significantly expressed. Compared with the control group, 3-dehydroepiandrosterone sulfate, oleic acid and linoleic acid were up-regulated, and gamma-muricholic acid was down-regulated in the NBp group; 3-dehydroepiandrosterone sulfate, 4-acetamidobutanoate and 1-hexadecanoyl-2-octadecadienoyl-sn-glyero-3-phosphocholine were up-regulated in the OBp group, whereas adenosine and 1-myristoyl-sn-glyero-3-phosphocholine were down-regulated; in the NH group, 1-palmitoyl-2-linoleoyl-sn-glycero-3-phosphocholine, phenol and 3-methoxytyramine were up-regulated, while pentadecanoic acid was down-regulated; in the OH group, NG,NG-dimethyl-L-arginine, 1-palmitoyl-sn-glycero-3-phosphocholine and monoethyl phthalate were up-regulated, while phloretin and glycine were down-regulated. The results showed that the children with hypertension had obvious disorders of lipid metabolism (especially in the overweight hypertension group), which led to the occurrence of hypertension. Additionally, the concentration of NO production-related NG, NG-dimethyl-L-arginine, was significantly increased, which may play an important role in H-type hypertension in children.

## 1. Introduction

Childhood hypertension was defined as three or more measurements of mean systolic and/or diastolic blood pressure greater than or equal to the 95th percentile of blood pressure in children of the same sex, age and height [1]. This is further subdivided into grade 1 (95th to the 99th percentile and 5 mmHg) and grade 2 (>99th percentile plus 5 mmHg) [2]. It is speculated that the overall morbidity rate of hypertension in children is about 2–5% [3], which shows a distinct escalating trend worldwide [4]. Elevated blood pressure in childhood can raise the risk of untimely death in adulthood [5] and seriously affect the normal growth and quality of life of children.

Previous studies have shown that genetic predisposition, body mass index (BMI), high salt intake, total cholesterol, triglyceride levels, irregular sleep, smoking and other factors are related to the occurrence of childhood hypertension [3,6,7]. Mutations in the serine-threonine (WNK) kinase gene cause inherited hyperkalemia and salt-sensitive hypertension through the WNK degradation pathway and WNK-SPAK-NCC phosphorylation cascade through the CUL3-KLHL3 E3 ligase complex [8]. Some other enzyme abnormalities, such as argininosuccinate lyase, phosphodiesterase 3A and class III histone deacetylase member SIRT6, also contribute to hypertension [9,10,11]. A study found that the lack of vitamin D in obese children can increase the secretion of renin and angiotensin II and the abnormal transcription of endothelial nitric oxide synthase (eNOS), resulting in hypertension [12]. Obesity can induce oxidative stress, hypoxia and inflammation, leading to the dysfunction of the fatty tissue around the blood vessels, abnormal regulation of perivascular-adipose-tissue-derived factors and disorder of the renin-angiotensin-aldosterone system (RAAS), which is involved in the occurrence of hypertension [13]. In addition, the immune system is crucial in the occurrence of hypertension. It can cross multiple organ systems, activate cytokines in the body, destroy endothelial function, induce vascular inflammation and lead to hypertension [14]. Elevated homocysteine (HHcy) is another common risk factor for hypertension. Plasma Hcy levels of 10 or more μmol/L were considered to indicate H-type hypertension in China’s “H-type hypertension diagnosis and therapy of expert consensus” in 2016. HHcy up-regulates the expression of interleukin-6 (IL-6) and nuclear factor-κB (NF-κB) p65/Rela, induces oxidative stress and inflammation, and leads to hypertension [15]. Boachie et al. found that low vitamin B12 levels can increase metabolic disorders and induce hypertension by affecting epigenetic mechanisms, such as DNA methylation, histone modifications and micro-RNA [16].

Metabolomics is a science based on nuclear magnetic resonance spectroscopy and mass spectrometry to study the changes in small molecule metabolites caused by internal and external stimuli, pathophysiological changes and gene mutations, including targeted and non-targeted metabolomics [17,18]. Previously, some studies have used the metabolomics method to explore the genetic modification of plants, the diagnosis of coronary artery disease, the marker of peroxisomal proliferation in animals, the apoptosis of tumors and so on [19]. At present, UHPLC-Q-TOF MS is the most commonly used method in non-targeted metabolomics. It uses two different chromatographic columns to comprehensively analyze small molecule metabolites in positive and negative ion modes, with high resolution, high throughput and high sensitivity [20,21]. Maria Averina et al. used the UHPLC-MS/MS method to analyze the relationship between serum perfluoroalkyl substances (PFAS) and hypertension in children and showed that PFAS concentration was positively correlated with the risk of hypertension in adolescents [22]. Other studies have also used non-targeted metabolomics methods to find that potential metabolites associated with hypertension in children mainly involve glycerophospholipids and amino acid metabolic pathways [23], and there is a strong correlation between the changes in the serum metabolic profiles of adolescent hypertensive patients and the molecular mechanism of hypertension [24].

However, the cause of hypertension in children is yet unknown. This work used ultra-high-pressure liquid chromatography and quadrupole time-of-flight mass spectrometry (UHPLC-Q-TOF MS) to study the differentially expressed metabolites in plasma samples from hypertensive children, to provide clues for the study of the mechanism of hypertension in children.

## 2. Materials and Methods

### 2.1. Samples Collection

The study’s subjects were 102 children with hypertension who were hospitalized at our hospital’s Department of Cardiology between June 2020 and May 2021, including normal body mass index and simple hypertension (NBp), overweight with simple hypertension (OBP), H-type hypertension with normal body mass index (NH), overweight with H-type hypertension (OH) (OBP and OH with BMI ≥ 24; NH and OH with plasma Hcy > 10 μmol/L), and 29 normotensive children. Briefly, participants with hypertension who met the following conditions were included in this study: (1) aged between 6 and 17 years; (2) mean systolic and/or diastolic blood pressure three or more times greater than or equal to the 95th percentile of blood pressure in children of the same sex, age and height [1]; (3) no antihypertensive drugs were used within 4 weeks of study initiation. Children with other organic heart diseases, secondary hypertension or aged <6 years were excluded. The study was approved by the Ethics Committee of the Chinese Capital Institute of Pediatrics (No: SHERLL2022017) and all guardians were fully informed of the purpose and procedure of this research. Written informed consent was obtained from guardians prior to study.

### 2.2. Preparation of Metabolite Extraction Samples

Peripheral blood samples from children with hypertension and the control group were collected in EDTA anticoagulant tubes. The blood samples were centrifuged at 3000× *g* 4 °C for 15 min, and the supernatant was transferred to fresh tubes and stored at −80 °C.

Samples were thawed slowly at 4 °C, appropriate amounts of samples were added into pre-cooled acetonitrile/methanol/water solution (2:2:1, *v*/*v*), mixed in vortex, ultrasonicated at low temperature for 30 min, −0 °C for 10 min, centrifuged at 14,000× *g* 4 °C for 20 min, and the supernatant was vacuum-dried. During mass spectrometry analysis, 100 μL acetonitrile aqueous solution (water: acetonitrile = 1:1, *v*/*v*) was added for reconstitution, vortexed and centrifuged at 14,000× *g* 4 °C for 15 min.

### 2.3. Chromatographic Conditions

The samples were separated by UHPLC (Agilent 1290 Infinity LC, Agilent Technologies, Santa Clara, CA, USA) HILIC column: the flow rate was 0.5 mL/min and the column temperature was 25 °C; mobile phase A: water +25 mM ammonium acetate +25 mM ammonia, mobile phase B: acetonitrile; injection volume: 2 μL. The following was the gradient elution process: 0–0.5 min, 95% B; 0.5–7 min, linear 95% to 65%; B changed linearly at the following time intervals: 7–8 min, linearly from 65% to 40%; 8–9 min, maintained at 40%; 9–9.1 min, linearly from 40% to 95%; 9.1–12 min, maintained at 95%. Throughout the analysis, the samples were placed in an automatic injector at 4 °C. The continuous analysis of samples was performed in random order to minimize the impact of instrument detection signal fluctuation. To monitor and assess the system’s stability and the validity of the experimental data, QC samples were added to the sample queue.

### 2.4. Q-TOF Mass Spectrometry Conditions

After the samples were separated by UHPLC system, primary and secondary mass spectrograms were performed by Triple TOF 6600 mass spectrometer (AB SCIEX, Framingham, MA, USA) in positive and negative spray ion source (ESI) modes. The following ESI source parameters were used: first-order mass-to-charge ratio detection range: 60–1000 Da; second-order ion mass-to-charge ratio detection range: 25–1000 Da; first-order mass spectrometry scan accumulation time: 0.2 s/spectra; second-order mass spectrometry scan accumulation time: 0.05 s/spectra; ion source temperature: 600 °C; spray voltage ± 5500 V (positive and negative modes); nebulizer-assisted heating gas 1 (Gas1): 60; assisted heating gas 2 (Gas2): 60; curtain gas: 30 psi; secondary mass spectrometry was obtained by data-dependent acquisition mode (IDA) and peak-intensity value screening mode; declustering voltage (DP): ±60 V (positive and negative modes); collision energy: 35 ± 15 eV; IDA set as follows: range of dynamic exclusion of isotope ions: 4 Da; 10 debris maps were collected per scan. All samples were assayed three times. An equivalent volume of a blank sample containing 100% acetonitrile was added to the sample queue at random for needle washing and column balancing while avoiding contamination between actual samples.

### 2.5. Data Processing

ProteoWizard MSConvert was used to convert the raw MS data (wiff.scan files) into MzXML files, and then imported to the free XCMS program for peak alignment, retention time correction and peak area extraction. The peak picking parameters are: peakwidth = c (10, 60), centWave m/z = 10 ppm, prefilter = c (10, 100). bw = 5, mzwid = 0.025, minfrac = 0.5 were used for peak grouping. CAMERA (Collection of Algorithms of Metabolite Profile Annotation) was used to annotate adducts and isotopes. At least a subset of the variables with non-zero measurements greater than 50% were kept among the ion features recovered by XCMS. Compound identification of metabolites was compared with various databases by precision m/z values (<10 ppm) and MS/MS spectra, including Kyoto Encyclopedia of Genes and Genomes (KEGG) (http://www.genome.jp/kegg/, accessed on 30 January 2023), the human database (http://www.hmdb.ca/, accessed on 5 January 2023; http://www.genome.jp/kegg/, accessed on 30 January 2023) and an internal database (Shanghai Applied Protein Technology Company, Shanghai, China).

After data processing and normalization, R package (ropls) was used for multivariate data analysis. This included orthogonal partial least squares discriminant analysis and principal component analysis on the pareto scale. The validity of the model was examined using the permutation test. In the orthogonal partial least squares discriminant analysis (OPLS-DA) model, the variable significance in the projection (VIP) value of each variable was calculated. In this experiment, strict OPLS-DA VIP > 1 and *p* < 0.05 were used as significant difference metabolite screening criteria. Single factor statistical analysis methods, including *t*-test/non-parametric test and fold-change analysis, were used to determine significant distinctions between the two groups of samples. Fold change > 1.5, *p* < 0.05 was considered up-regulation. Fold change < 0.67, *p* < 0.05 was considered down-regulation.

For further investigation of the association between metabolites and samples, heatmaps were produced using the pheatmap R package (Kolde R. pheatmap: Pretty Heatmaps; r package version 1.0.8; https://cran.r-project.org/web/packages/pheatmap/index.html, accessed on 30 January 2023). To study the potential correlation between metabolites, we calculated the Spearman correlation test between each two metabolites to conduct correlation-based network analysis. Finally, only correlations of |r| > 0.8 and *p* < 0.05 were considered and Cytoscape was used to visualize the correlation-based network. Correlation analysis can measure the metabolic proximities between markedly different metabolites (VIP > 1, *p* < 0.05), allowing for a further understanding of the mutual adjustment relationship between metabolites in the process of biological state changes.

The metabolic patterns of metabolites under different experimental conditions were determined by cluster analysis. Then, KEGG was utilized to analyze the relationship between compounds in metabolic pathways. Fisher’s precision test was utilized to calculate and analyze significant levels of metabolite enrichment in each pathway. The smaller the *p* value, the higher the significant level of the pathway.

### 2.6. Statistical Analysis

All statistical analyses were performed using SPSS 22.0 software and GraphPad Prism 9.0 software. The normality of quantitative variables was established by Kolmogorov–Smirnov test. The statistical validity of all experimental condition differences was established by one-way analysis of variance (ANOVA), and repeated measures of analysis of variance were performed for intra-group differences. Chi-square test or Fisher’s exact test was used for pairwise comparison. Sex, age, BMI and hemodynamic were tested by independent Student’s t test, and the parameters were expressed by mean and standard deviation. Plasma Hcy levels were calculated by Mann–Whitney U test, and parameters were expressed as medians and quartiles. The significance level was established at 5%.

## 3. Results

### 3.1. Subject Characteristics

To find the related plasma differential metabolites in children with hypertension, sera of 38 children with normal body mass index and simple hypertension (the NBp group), 8 children overweight with simple hypertension (the OBp group), 37 children with normal body mass index and H-type hypertension (the NH group), 19 children overweight with H-type hypertension (the OH group) and 29 normal controls (the C group) were collected. Table 1 showed the sex, age, sSBP, sDBP, Hcy and BMI of the hypertension group and the control group. Compared with the C group, sSBP and sDBP in the hypertension group were significantly elevated, *p* < 0.001. In addition, the Hcy levels in the NH and OH groups were markedly higher than that in the C, NBp and OBp groups, and the differences were statistically significant. The BMI levels in the OBp and OH groups were markedly different from those of the control group (29.00 ± 3.18/29.57 ± 4.03 vs. 20.62 ± 5.04; *p* < 0.001). There was no statistical significance in BMI among the control, NBp and NH groups. Next, we used these samples for metabolomics analysis.

### 3.2. Differential Metabolites between Hypertension Groups and Normal Control

**Figure 1:**
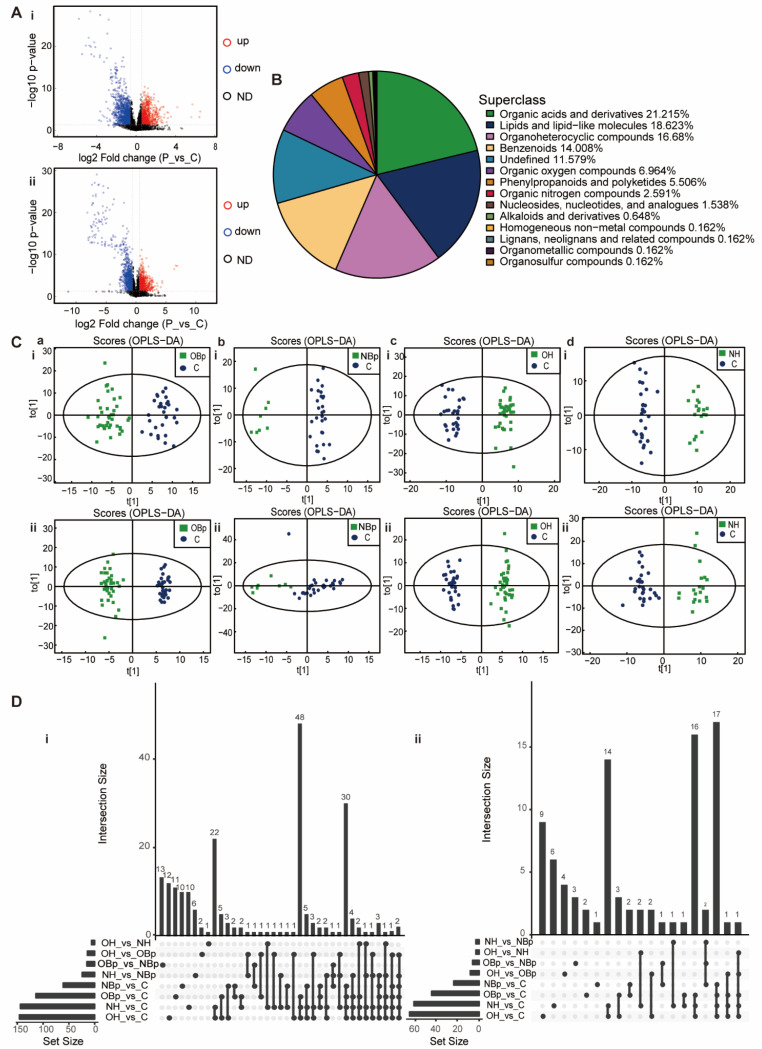
Principal component analysis of the metabolites. (**A**) Volcanic maps of positive ion mode (i) and negative ion mode (ii). Red dots on behalf of up-regulated metabolites satisfying FC > 1.5, *p* < 0.05, blue dots on behalf of down-regulated metabolites satisfying FC < 0.67, *p* < 0.05, and black points on behalf of metabolites without a difference. P: hypertension group; C: control group. (**B**) The pie chart shows 14 superclasses. The top four are organic acids and derivatives (21.215%), lipids and lipid-like molecules (18.623%), organoheterocyclic compounds (16.68%), and benzenoids (14.008%). The others were organic oxygen compounds (6.964%), phenylpropanoids and polyketides (5.506%), organic nitrogen compounds (2.591%), nucleosides nucleotides and analogues (1.538%), alkaloids and derivatives (0.648%), homogeneous non-metal compounds (0.162%), lignans, neolignans and related compounds (0.162%), organometallic compounds (0.162%), organohalogen compounds (0.162%) and 11.579% of metabolites were undefined. (**C**) (i,ii) The positive ion mode (i) and negative ion mode (ii) OPLS-DA analysis. Green points represent each biological repetition in group OBp (a), group NBp (b), group OH (c), and group NH (d). The ellipse represents 95% confidence interval and blue points represent each biological repetition in the control group. The t [1] is the score of the samples on the first principal component to distinguish between groups, and the to [1] is the score of the samples on the second principal component to reflect the intra-group variation. (**D**) The Venn diagram shows the overlap between groups of obviously different metabolites. Comparative analysis of all differential metabolites between the groups. The bar chart on the left shows the amounts of different compounds contained in each comparison group. The lower intersection points refer to the corresponding comparison group names on the left. The connection between longitudinal points represents the intersection between corresponding data sets. The bar chart at the top corresponds to the number of metabolites shared across the comparison groups. (i) Positive ion mode; (ii) negative ion mode.

To study the differences in compounds in children with hypertension at the overall level, we first analyzed the plasma samples of the hypertension group and the control group by HPLC-Q-TOF MS. A total of 480 negative ion mode ions and 755 positive ion mode ions were identified. OPLS-DA results showed that the hypertension group was significantly separated from the control group with a high degree of aggregation within the group and the difference between the groups was statistically significant (Figure S1A). Among them, there were 193 total differential metabolites, 85 increased metabolites and 108 decreased metabolites (Figure 1A). A total of 1235 metabolites were identified, belonging to 14 superclasses. It can be seen that organic acids and derivatives (21.215%) accounted for the largest proportion, the second superclass is lipids and lipid-like molecules (18.623%), and organoheterocyclic compounds (16.68%) are ranked third (Figure 1B). It is suggested that organic acids and derivatives and lipids and lipid-like molecules are critical in the pathogenesis of hypertension in children.

Subsequently, we screened out some differential metabolites that were most closely related to hypertension in children according to VIP > 1, *p* < 0.05, as shown in Table 2. 3-dehydroepiandrosterone sulfate (DHEAS), gamma-muricholic acid, 2-hydroxyisocaproic acid and adrenosterone belong to lipids and lipid-like molecules, among which DHEAS, 2-hydroxyisocaproic acid and adrenosterone were up-regulated, and gama-muricholic acid was down-regulated. DL-isoleucine, glycine, NG, NG-dimethyl-L-arginine (ADMA) belong to organic acids and derivatives, of which DL-isoleucine and ADMA are up-regulated and glycine is down-regulated. In addition, there are other types of metabolites, such as dopamine, monoethyl phthalate, 3-methoxytyramine, which were up-regulated, and adenosine, acetylcholine, adenosine 3′, 5′-cyclic monophosphate (cAMP), and phloretin were down-regulated. The above metabolites have changed significantly in children with hypertension, indicating that the changes in these metabolites play an important role in the molecular mechanism of hypertension in children. Due to the complex pathogenic factors of hypertension in children, we have carried out further analysis for the hypertension group in children.

### 3.3. Differential Metabolites in 4 Hypertension Groups

We divided the hypertension group into four groups: NBp, OBp, NH and OH for analysis to explore the specific role of metabolites. QC samples were tightly clustered together in positive and negative ion modes (Figure S1B), indicating good repeatability of the experiment. The OBp, NBp, OH and NH groups were significantly separated from the control group (Figure 1C), indicating significant differences in serum metabolites between the five groups and good model stability. The permutation test improved the analytical power and validity of the OPLS-DA analysis (Figure S1D). Compared with other comparison groups, there were 47 obviously diverse metabolites in the overlap of OBp/NBp/OH/NH vs. C four groups, 64 obviously different metabolites in the overlap of OBp/OH/NH vs. C three groups, and 36 distinctly diverse metabolites in the overlap of OH/NH vs. C two groups. There were 21 significantly different metabolites that were only changed in OH vs. C (Figure 1D). It can be seen that the number of unique metabolites increased significantly in the hypertension group. Heat maps showed that the trend of most metabolites expression in the hypertensive group was reversed from that in the control group. For example, in the NH group (Figure S1C,d), 3,4-Dihydroxyhydrocinnamic acid, ADMA, 3-hydroxykynurenine, adrenosterone, cytosine, phenylalanine, glutamine and other metabolites were up-regulated; evodiamine, L-homocitrulline, acetylcysteine, His-ser, cAMP, gamma-muricholic acid, etc., were down-regulated. At the same time, based on the metabolite–enzyme–gene network diagram, we found that the metabolism of methionine and cysteine is regulated by various enzymes and genes, which is a complex network diagram, and the metabolism of various amino acids (Figure S2). Since methionine metabolism is closely related to plasma HHcy, we focused on the enzymes in the methionine metabolic pathway in order to find a breakthrough in the mechanism of H-type hypertension.

**Figure 2:**
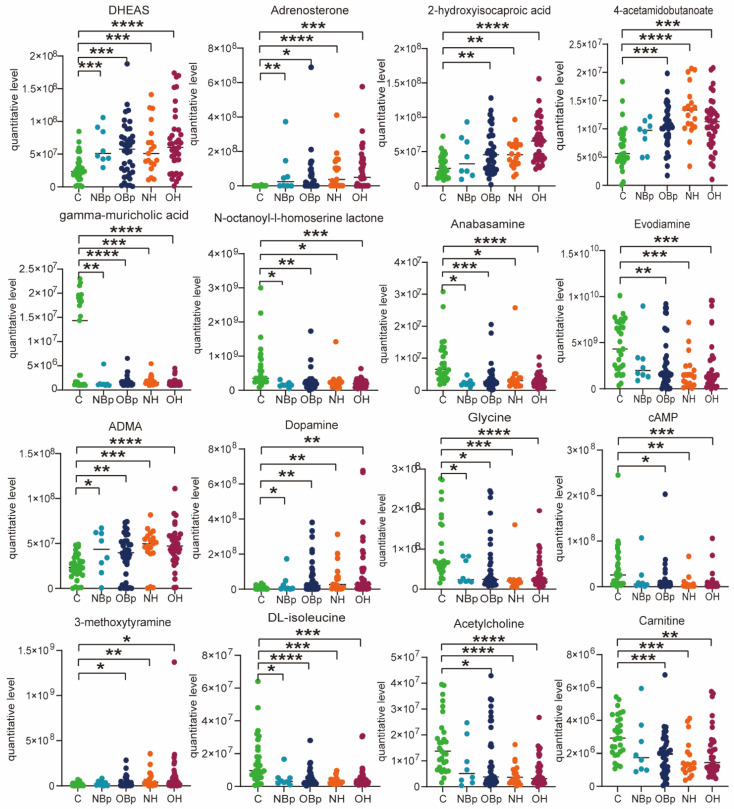
Scatter diagram of hypertension-related metabolites. DHEAS: 3-dehydroepiandrosterone sulfate. ADMA: NG, NG-dimethyl-L-arginine. cAMP: adenosine 3′,5′-cyclic monophosphate. Comparison of differential metabolites among the five groups. (* *p* < 0.05, ** *p* < 0.01, *** *p* < 0.001, **** *p* < 0.0001).

Next, the common differential metabolite levels in the four groups were compared, mainly divided into lipid and non-lipid substances. We drew the corresponding scatter plots as shown in Figure 2. Obviously, the lipid metabolites DHEAS, adrenosterone, 2-hydroxyisocaproic acid and gamma-muricholic acid increased/decreased most significantly in the OH group, followed by the OBp group, compared with the control group. It can be seen that the lipid metabolism disorder is more serious when overweight combined with high Hcy. Similarly, n-octanoyl-l-homoserine lactone, anabasamine, DL-isoleucine and carnitine were more significantly altered in the OBp and OH groups. In addition, 4-acetamidobutanoate, evodiamine, ADMA, dopamine, glycine, cAMP and acetylcholine changed more significantly in the NH and OH groups, especially ADMA, which increased most significantly in the OH group (*p* < 0.0001). The results suggested that all hypertensive children had different degrees of lipid metabolism disorder, especially in the OBp and OH groups when the increase/decrease in lipids and lipid-like molecules was more significant. However, some metabolites are specific to a certain group, so we analyzed each hypertension group separately.

**Figure 3:**
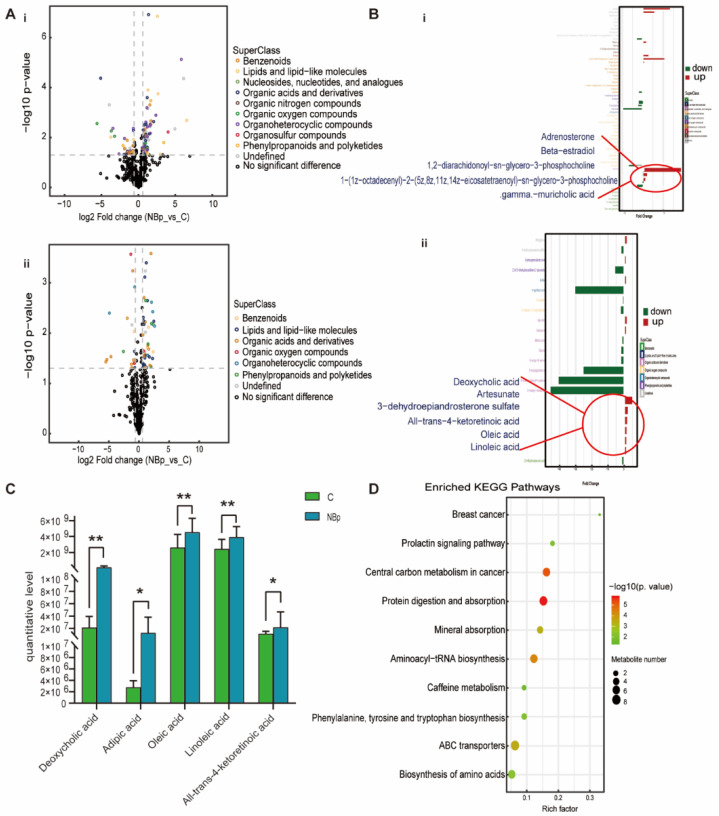
Analysis of differential metabolites between NBp vs. C. (**A**) Volcanic maps of positive (i) and negative (ii) ion modes. Points on the right half of volcano plot represent up-regulated metabolites satisfying FC > 1.5, *p* < 0.05, and points on the left half represent down-regulated metabolites satisfying FC < 0.67, *p* < 0.05, and black points on behalf of non-differential metabolites. (**B**) (i,ii) Bar graphs of the positive and negative ion modes. The abscissa indicates the fold change (FC). Red bars on behalf of up-regulated metabolites satisfying FC > 1, *p* < 0.05, green bars on behalf of down-regulated metabolites satisfying FC < 1, *p* < 0.05. The ordinate represents significantly distinctive metabolites. Local enlarged view for lipids and lipid-like molecules. (**C**) Differential metabolites unique to the NBp group. (* *p* < 0.05, ** *p* < 0.01). (**D**) KEGG pathway enrichment bubble diagram. The vertical axis represents the KEGG metabolic pathway, and the horizontal axis represents the rich factors (the ratio of the number of differential metabolites to the number of annotated metabolites in this pathway). The redder the circle color, the smaller the *p*-value of enrichment analysis and the higher the channel significance level.

For the NBp group, it can be clearly seen that benzenoids, lipids and lipid-like molecules, organoheterocyclic compounds and organic acids and derivatives have different degrees of up-regulation and down-regulation (Figure 3A). The histogram visually shows the fold change in the differential metabolites specifically contained in the superclass, where we highlight lipids and lipid-like molecules (Figure 3B). We found that adrenosterone, 1,2-diarachidonoyl-sn-glycero-3-phosphocholine, deoxycholic acid, artesunate, DHEAS, all-trans-4-ketoretinoic acid, oleic acid and linoleic acid were up-regulated; 1-(1z-octadecenyl)-2-(5z,8z,11z,14z-eicosatetraenoyl)-sn-glycero-3-phosphocholine and gamma-muricholic acid were down-regulated. In addition, deoxycholic acid, adipic acid, oleic acid, linoleic acid, and all-trans-4-ketoretinoic acid were specifically associated with the NBp group (Figure 3C). The enrichment of the KEGG pathway showed the top 20 metabolic pathways with the most significant differences, mainly in protein digestion and absorption, aminoacyl-tRNA biosynthesis, ABC transporters, biosynthesis of amino acids and other pathways (Figure 3D). All the results of the NBp group showed that children with normal BMI had lipid metabolism disorders, suggesting that dyslipidemia is critical in the occurrence of hypertension in children.

**Figure 4:**
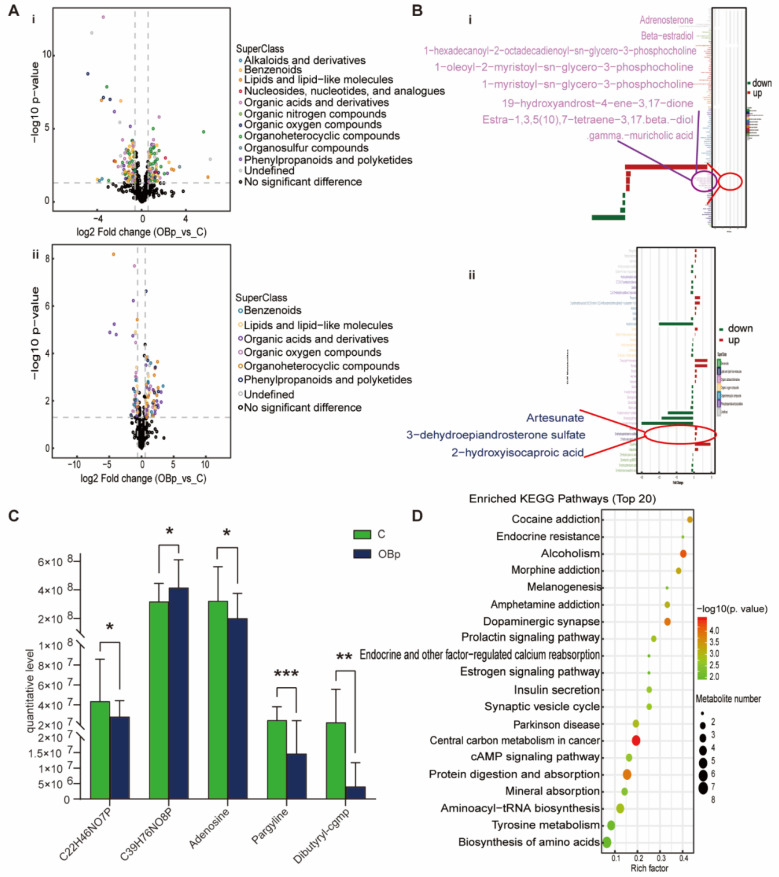
Analysis of differential metabolites between OBp vs. C. (**A**) Volcanic maps of positive (i) and negative (ii) ion modes. Points on the right half of volcano plot represent up-regulated metabolites satisfying FC > 1.5, *p* < 0.05, and points on the left half represent down-regulated metabolites satisfying FC < 0.67, *p* < 0.05, and black points on behalf of non-differential metabolites. (**B**) (i,ii) Bar graphs of the positive and negative ion modes. The abscissa indicates the fold change (FC). Red bars on behalf of up-regulated metabolites satisfying FC > 1, *p* < 0.05, green bars on behalf of down-regulated metabolites satisfying FC < 1, *p* < 0.05. The ordinate represents significantly distinctive metabolites. Local enlarged view for lipids and lipid-like molecules. (**C**) C22H46NO7P: 1-myristoyl-sn-glycero-3-phosphocholine. C39H76NO8P: 1-hexadecanoyl-2-octadecadienoyl-sn-glycero-3-phosphocholine. Differential metabolites unique to the to the OBp group. (* *p* < 0.05, ** *p* < 0.01, *** *p* < 0.001). (**D**) KEGG pathway enrichment bubble diagram. The vertical axis represents the KEGG metabolic pathway, and the horizontal axis represents the rich factors (the ratio of the number of differential metabolites to the number of annotated metabolites in this pathway). The redder the circle color, the smaller the *p*-value of enrichment analysis and the higher the channel significance level.

For the OBp group, phenylpropanoids and polyketides, organic acids and derivatives, benzenoids, lipids and lipid-like molecules and organoheterocyclic compounds were up-regulated and down-regulated to varying degrees (Figure 4A). In lipids and lipid-like metabolites, adrenosterone, beta estradiol, 1-hexadecanoyl-2-octadecadienoyl-sn-glycero-3 phosphocholine, 1-oleoyl-2-myristoyl sn-glycero-3 phosphocholine, artesunate, DHEAS, and 2-hydroxyisocaproic acid were up-regulated; 1-myristoyl-sn-glycero-3-phosphocholine, 19-hydroxyandrost-4-ene-3,17-dione, estra-1,3,5(10),7-tetraene-3,17beta-diol, and gamma-muricholic acid were down-regulated (Figure 4B). The metabolites specifically associated with the OBp group were 1-myristoyl-sn-glycero-3-phosphocholine, 1-hexadecanoyl-2-octadecadienoyl-sn-glycero-3-phosphocholine, adenosine, pargyline, and dibutyryl-cgmp (Figure 4C). The KEGG pathway was mainly enriched in endocrine resistance, dopaminergic synapse, endocrine- and other factor-regulated calcium reabsorption, insulin secretion, cAMP signaling pathway, protein digestion and absorption, aminoacyl-tRNA biosynthesis, biosynthesis of amino acids and other pathways (Figure 4D). Compared with the NBp group, the types of lipid metabolites in the OBp group increased, and the change trend was more significant, indicating that weight gain had a significant regulatory effect on these metabolites. Moreover, the KEGG pathway is also clustered in the endocrine- and other factor-regulated calcium reabsorption pathways, which may have a potential effect on calcium in overweight hypertensive children, and the specific role needs to be further verified. In brief, the OBp group showed more differences in lipids, and the trend is more significant, suggesting that high BMI is directly associated with the occurrence of hypertension in children.

**Figure 5:**
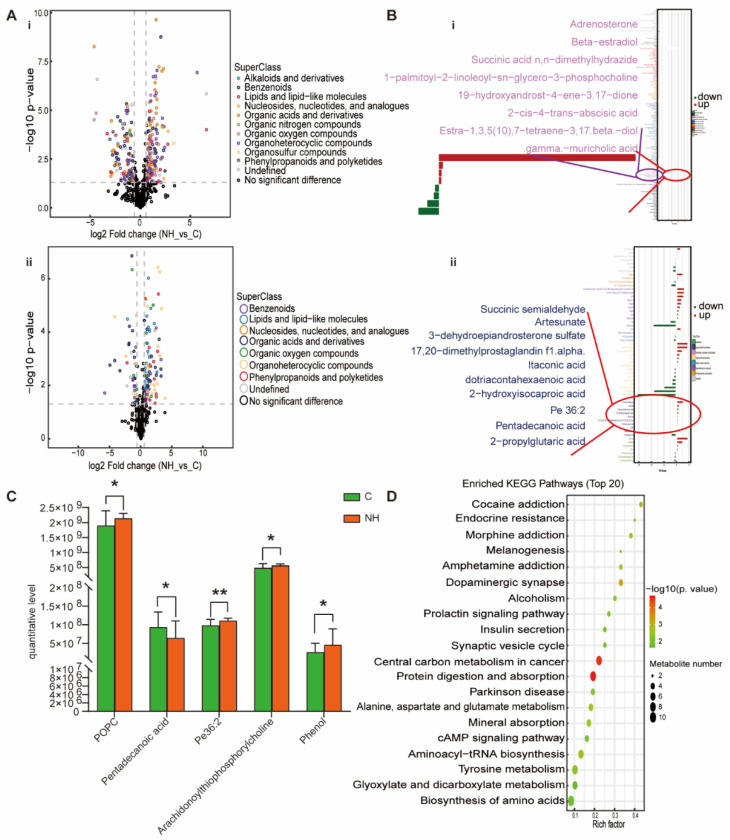
Analysis of differential metabolites between NH vs. C. (**A**) Volcano plot of positive (i) and negative (ii) ion modes. Points on the right half of volcano plot represent up-regulated metabolites satisfying FC > 1.5, *p* < 0.05, and the points on the left half represent down-regulated metabolites satisfying FC < 0.67, *p* < 0.05, and black points on behalf of non-differential metabolites. (**B**) (i,ii) Bar graphs of the positive and negative ion modes. The abscissa indicates the fold change (FC). Red bars on behalf of up-regulated metabolites satisfying FC > 1, *p* < 0.05, green bars on behalf of down-regulated metabolites satisfying FC < 1, *p* < 0.05. The ordinate represents significantly distinctive metabolites. Local enlarged view for lipids and lipid-like molecules. (**C**) POPC: 1-palmitoyl-2-linoleoyl-sn-glycero-3-phosphocholine. Differential metabolites unique to the to the NH group. (* *p* < 0.05, ** *p* < 0.01). (**D**) KEGG pathway enrichment bubble diagram. The vertical axis represents the KEGG metabolic pathway, and the horizontal axis represents the rich factors (the ratio of the number of differential metabolites to the number of annotated metabolites in this pathway). The redder the circle color, the smaller the *p*-value of enrichment analysis and the higher the channel significance level.

For the NH group, organoheterocyclic compounds, organic acids and derivatives, benzenoids, lipids and lipid-like molecules, organic oxygen compounds and nucleosides, nucleotides, and analogues were up-regulated and down-regulated to varying degrees (Figure 5A). In locally amplified lipids and lipid-like metabolites, adrenosterone, beta-estradiol, succinic acid n,n-dimethylhydrazide, 1-palmitoyl-2-linoleoyl-sn-glycero-3-phosphocholine, succinic semialdehyde, artesunate, DHEAS, 17, 20-dimethylprostaglandin f1α, itaconic acid, dotriacontahexaenoic acid, 2-hydroxyisocaproic acid, and Pe 36:2 were up-regulated; 19-hydroxyandrost-4-ene-3, 17-dione, 2-cis-4-trans-abscisic acid, estra-1, 3, 5 (10), 7-tetraene-3, 17 beta-diol, gamma-muricholic acid, pentadecanoic acid, and 2-propylglutaric acid were down-regulated (Figure 5B). Moreover, the NH-group-specific lipid metabolites were 1-palmitoyl-2-linoleoyl-sn-glyero-3-phosphocholine, pentadecanoic acid, Pe36:2, and arachidonoylthiophosphorylcholine (Figure 5C). Additionally, compared with the NBp and OBp groups, the changes in adrenosterone, 4-acetamidobutanoate, gamma-muricholic acid, evodiamine, ADMA, glycine, DL-isoleucine and acetylcholine in the NH group were more significant (Figure 2). We found that not only lipid metabolites, such as pentadecanic acid, adrenal ketone, Pe 36:2, but other non-lipid metabolites, such as picediamine, DL-isoleucine, and glycine, also had significant changes in the NH group, which were more serious than those in the NBp and OBp groups, indicating that the simple increase in HCY level could lead to the disorder of various metabolite levels in the body. Finally, the KEGG pathway was mainly enriched in endocrine resistance, dopaminergic synapse, insulin secretion, protein digestion and absorption, alanine, aspartate and glutamate metabolism, cAMP signaling pathway, tyrosine metabolism, biosynthesis of amino acids and other pathways (Figure 5D). We found that compared with children with simple hypertension or simple obesity hypertension, children with high Hcy have more types of plasma differential metabolites and more significant changes, indicating that the increase in Hcy level alone cannot only cause lipid metabolism disorders, but can also lead to changes in various metabolites through other ways involved in the development of hypertension in children.

**Figure 6:**
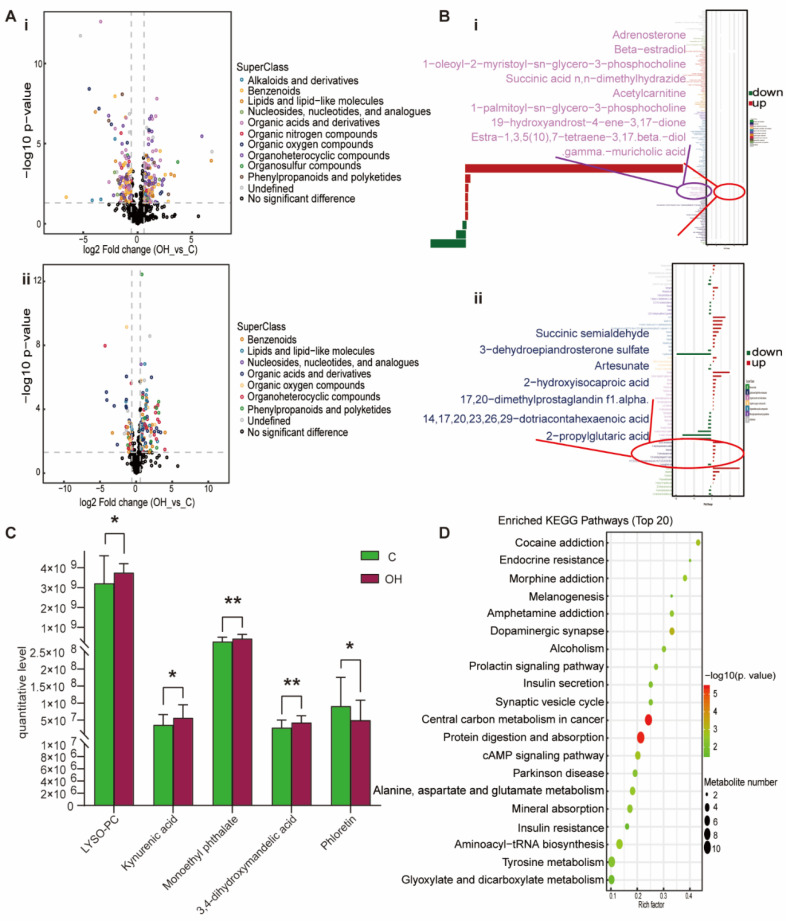
Analysis of differential metabolites between OH vs. C. (**A**) Volcano plot of positive (i) and negative (ii) ion modes. Points on the right half of volcano plot represent up-regulated metabolites satisfying FC > 1.5, *p* < 0.05, and the points on the left half represent down-regulated metabolites satisfying FC < 0.67, *p* < 0.05, and black points on behalf of non-differential metabolites. (**B**) (i,ii) Bar graphs of the positive and negative ion modes. The abscissa indicates the fold change (FC). Red bars on behalf of up-regulated metabolites satisfying FC > 1, *p* < 0.05, green bars on behalf of down-regulated metabolites satisfying FC < 1, *p* < 0.05. The ordinate represents significantly distinctive metabolites. Local enlarged view for lipids and lipid-like molecules. (**C**) LYSO-PC: 1-palmitoyl-sn-glycero-3-phosphocholine. Differential metabolites unique to the to the OH group. (* *p* < 0.05, ** *p* < 0.01). (**D**) KEGG pathway enrichment bubble diagram. The vertical axis represents the KEGG metabolic pathway, and the horizontal axis represents the rich factors (the ratio of the number of differential metabolites to the number of annotated metabolites in this pathway). The redder the circle color, the smaller the *p*-value of enrichment analysis and the higher the channel significance level.

For the OH group, lipids and lipid-like metabolites, benzenoids, organic acids and derivatives, phenylpropanoids and polyketides and organoheterocyclic compounds were up-regulated to varying degrees (Figure 6A). Adrenosterone, beta-estradiol, 1-oleoyl-2-myristoyl-sn-glycero-3-phosphocholine, succinic acid n,n-dimethylhydrazide, acetylcarnitine, 1-palmitoyl-sn-glycero-3-phosphocholine, succinic semialdehyde, 3-dehydroepiandrosterone sulfate, artesunate, 2-hydroxyisocaproic acid, 17, 20-dimethylprostaglandin f1α and dotriacontahexaenoic acid were up-regulated in lipids and lipid like molecules; 19-hydroxyandrost-4-ene-3, 17-dione, estra-1, 3, 5 (10), 7-tetraene-3, 17 beta-diol, gamma-muricholic acid and 2-propylglutaric acid were down-regulated (Figure 6B). The metabolites specifically associated with the OH group were 1-palmitoyl-sn-glycero-3-phosphocholine, kynurenic acid, monoethyl phthalate, 3,4-dihydroxymandelic acid and phloretin (Figure 6C). The KEGG pathway was mainly enriched in endocrine resistance, dopaminergic synapse, insulin secretion, protein digestion and absorption, cAMP signaling pathway, alanine, aspartate and glutamate metabolism, aminoacyl-tRNA biosynthesis and tyrosine metabolism (Figure 6D). Obviously, the OH group has the largest variation in differential metabolites, and the enrichment pathway is more extensive. It is important to note that the OH group is enriched in the insulin resistance pathway, and Figure S3 displays its KEGG pathway. Based on the above results, we found that the OH group not only had lipid metabolism disorders, but also had insulin resistance. So, it has been shown that being overweight and having a high Hcy level can lead to insulin resistance and hasten the onset of hypertension. Compared with the other three groups, the changes in Lipids and lipid-like molecules, such as DHEAS, 2-hydroxyisocaproic acid and gamma-muricholic acid in the OH group, were particularly significant. In addition, other non-lipid molecules, such as anabasamine, ADMA, glycine and acetylcholine, also showed the most significant changes in the OH group (Figure 2). It indicated that the effect of high BMI combined with high Hcy on hypertension in children is more serious than that of simple high BMI or simple high Hcy alone.

To sum up, the quantitative concentrations of lipids and lipid-like metabolites, such as DHEAS, 4-acetamidobutanoate, adrenosterone, oleic acid, linoleic acid, pentadecanoic acid and Pe36:2, have changed significantly in the children with hypertension, especially in the overweight hypertension group, resulting in primary dyslipidemia, which is involved in the development of hypertension in children. Secondly, other metabolites related to hypertension, such as ADMA, dopamine, adenosine, 3-methoxytyramine, glycine, etc., have also undergone significant changes in children with hypertension. In particular, ADMA is significantly increased in children with H-type hypertension, which may be very critical in children with H-type hypertension.

## 4. Discussion

This study used non-targeted metabolomics to detect subtle changes in plasma metabolomics in children with simple and H-type hypertension and explore potential molecular mechanisms of related metabolites leading to hypertension, to open up new doors for the early detection and cure of children with hypertension. We found that all hypertensive children had abnormal lipid metabolism, with DHEAS, 1-oleoyl-2-myristoyl-sn-glycero-3-phosphocholine, adrenosterone, 2-hydroxyisocaproic acid and 4-acetamidobutanoate up-regulated, whereas gamma-muricholic acid, estra-1, 3, 5 (10), 7-tetraene-3, 17-beta-diol and 2-propylglutaric acid were down-regulated. In addition, we found that the quantitative concentrations of ADMA, glycine, 3-methoxytyramine, dopamine, DL-isoleucine, evodiamine, acetylcholine and other metabolites were significantly altered in children with hypertension. Elevated dopamine and decreased adenosine are likely to impact the cAMP-PKA signaling pathway (Figure S4). Hypertension is caused by diminished renal sodium excretion as well as inhibition of the Na+/K+/ATPase pump and Na+/H+ exchanger. In addition, the expression patterns of metabolites belonging to the same group were similar (Figure S1C), but just a few patient samples from the control group fell into the experimental group. The clinical characteristics of these samples included symptoms of dizziness and syncope. There may be a correlation between the metabolites in these control children and metabolites in children with hypertension, and a detailed explanation for this finding needs further study.

In this investigation, lipids and lipid-like metabolites, such as DHEAS, adrenosterone, 2-hydroxyisocaproic acid, 4-acetamidobutanoate and gamma-muricholic acid, showed significant changes in the four groups of hypertensive children and were associated with the emergence of childhood hypertension. Circulating DHEAS is a form of dehydroepiandrosterone [25], which is mainly produced in the adrenal gland and reaches a maximum plasma level between 15 and 25 years of age. In human blood, it is the steroid hormone that is most prevalent. In the Turkish population, obese men had higher DHEAS levels, larger waist circumference and lower serum insulin levels than obese women [26]. Serum DHEAS levels in children were reported to be directly associated with body and trunk fat, and positively associated with insulinemia [27]. Additionally, when compared to children who are not fat, DHEAS has a significant impact on the risk of atherosclerosis, with a higher risk of hypertension and a danger that lasts into adulthood [25]. Studies have shown that variations in systolic and diastolic blood pressure were positively connected with DHEAS levels in women, but not in males, so there may be sex differences [28]. However, some studies have shown that low levels of DHEAS were beneficial to obesity and cardiovascular disease [25], and can reduce vascular smooth muscle proliferation and increase apoptosis [29]. In the elderly, DHEAS can also be used as a preventive drug for cardiovascular disease [30], which seems to contradict our research. However, for children, DHEAS may be related to sex, diet, genetics, lifestyle and other factors, and its specific temporal role needs further study. 2-hydroxyisocaproic acid has antibacterial effects [31]. The current study showed that 2-hydroxyisocaproic acid was more abundant in the overweight hypertension groups (OBp and OH), but its relationship with hypertension in obese children has not been reported. 4-acetamidobutanoate is a product of the urea cycle, and its levels increase with renal dysfunction. 2-hydroxyisocaproic acid is also related to lysine degradation and phenylalanine metabolism [32]. Among the four groups of children with hypertension, the average level of 4-acetamidobutanoate was relatively high in the NH group and may reflect the different effects of lipid metabolism and Hcy metabolism. Finally, we speculate that in the overweight hypertension group, the up-regulation/down-regulation of lipids and lipid-like metabolites may affect the accumulation of hormones in adipocytes, resulting in central obesity, leading to metabolic disorders involved in the occurrence of hypertension. These lipids and lipid-like metabolites may not have a significant impact on how children acquire hypertension, but primary dyslipidemia brought on by lipid metabolism disorders is a major factor in how obese people develop atherosclerosis and cardiovascular disease [33]. Adipose tissue is an active endocrine system, and RAAS hormones are secreted directly by fat cells. Studies have shown that RAAS activation is associated with obesity and hypertension, dyslipidemia and pro-inflammatory status. RAAS activation may also be another important mechanism of hypertension in obese patients [34].

In addition, the current study showed that there are some lipid and lipid-like molecules or non-lipid substances that only change in a certain group of children with hypertension. Studies have revealed that non-esterified fatty acids, in particular oleic acid, have a synergistic mitogenic effect with angiotensin II on the growth and proliferation of rat aortic smooth muscle cells, and can accelerate vascular remodeling, which has been considered a risk factor for atherosclerosis [35,36]. Oleic acid can induce the production of reactive oxygen species in endothelial cell mitochondria and reduce the activity of eNOS [37]. Linolenic acid can induce insulin resistance in rats [38]. Elevated levels of oleic and linoleic acids can affect blood pressure by regulating the sensitivity of Na+K+-ATPase to cardiac glycosides [39]. In the NBp group, we found higher levels of oleic and linoleic acids. Although the BMI of these children remained within the normal range, if the criteria for defining obesity in children were used, some children had already developed obesity. It is possible that simple blood lipids were abnormal, or there was a tendency towards obesity, which was extremely detrimental to the health of children with hypertension. Elevated deoxycholic acid levels can induce vascular calcification, which is connected with cardiovascular disease mortality. Deoxycholic acid is a biomarker of vascular calcification in patients with chronic kidney disease [40,41]. In addition, adenosine can induce hypertensive renal vasodilation by stimulating adenosine receptors [42]. Adenosine levels were decreased in the OBp group, which may further enhance children’s development of hypertension. There is a positive correlation between phenols and hypertriglyceridemia and hypertension [43]. Phthalates can cause oxidative stress and pro-inflammatory responses, which are closely connected to elevated blood pressure [44]. Furthermore, increased pentadecanoic acid intake was negatively correlated with liver fat, cardiovascular disease and type 2 diabetes; that is, low levels of pentadecanoic acid were not conducive for children with hypertension [45]. However, whether increased pentadecanoic acid intake is connected with an increased risk of cardiovascular disease needs further research [46]. It has also been found that phloretin can reduce the inflammatory injury and fibrosis of mouse cardiomyocytes by restoring silent information regulator 2 homolog 1 expression, and has a strong protective effect on cardiomyocytes [47]. These substances may play different roles in the four groups of hypertension, but all of them are extremely unfavorable for the cure of pediatric hypertension. On the basis of changes found in levels of these metabolites, finding the most critical mechanism of action may provide the best therapeutic avenue for these children.

**Figure 7:**
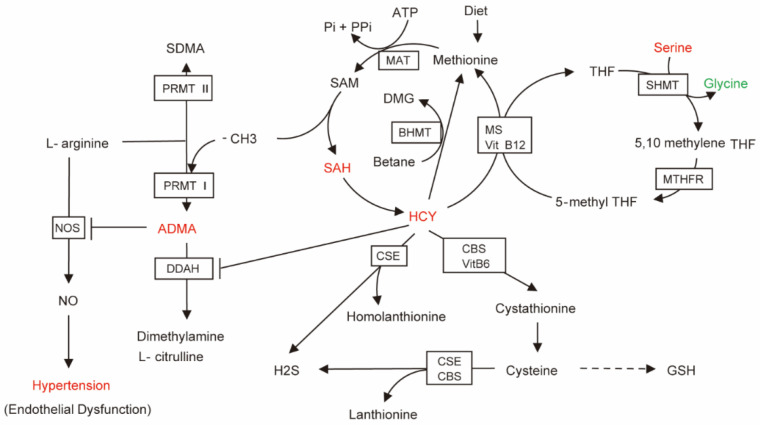
Brief diagram of HCY metabolic pathways related to hypertension. The arrow direction is the direction of metabolite generation. The red metabolites represent up-regulated metabolites satisfying FC > 1, *p* < 0.05, green metabolite represents down-regulated metabolite satisfying FC < 1, *p* < 0.05. Enzymes acting on the pathway are shown in the boxes.

ADMA is a protein methyltransferase-produced endogenous NOS inhibitor [48]. Hcy and ADMA are well-known risk markers for cardiovascular disease, and are generally elevated in patients with hypertension [49]. It is well known that NO is the strongest endogenous vasodilator in the body. However, high concentrations of ADMA and homocysteine can inhibit NOS, leading to NO synthesis disorders that change vascular endothelial function and then hypertension [49,50]. In addition, the enzyme dimethylaminohydrolase that regulates ADMA degradation can be inhibited by elevated Hcy, resulting in an increase in ADMA concentration. Hcy can also induce oxidative stress, resulting in higher ADMA levels, forming a vicious cycle [51,52,53]. In our research, children with hypertension had considerably higher ADMA levels, especially in children with H-type hypertension. Therefore, we proposed a potential mechanism for HHcy leading to hypertension, as shown in a metabolic pathway diagram (Figure 7), which demonstrates that ultimately NO production may be blocked, leading to NO downstream cGMP-PKG signaling pathway abnormalities (Figure S4C), vascular smooth muscle contraction and the emergence of hypertension. It has also been shown in rat models that serum ADMA can induce eNOS decoupling through substrate reduction, increasing the production of superoxide radicals and inhibiting the formation of NO in blood vessels [54]. Therefore, targeting ADMA may be a strategy for treating hypertension. In addition, elevated levels of Hcy alone were closely related to cardiovascular disease, and may lead to oxidative stress, endothelial cell damage and atherosclerosis [55]. Endogenous H2S levels can decrease with increasing Hcy levels, resulting in increased blood pressure via activation of RAAS and extracellular-signal-regulated kinase 1/2 signaling pathways [56]. HHcy is also a factor affecting insulin resistance, because HHcy can mediate the expression and secretion of resistin as well as certain inflammatory factors [57]. Therefore, in the OH group of the current study, KEGG pathway analysis showed factors gathered in the insulin resistance pathway, and additional studies should focus on the connection between HHcy and insulin resistance. In conclusion, the treatment of hypertensive children with HHcy should involve weight control and also the maintenance of plasma Hcy concentrations within the normal range.

Dopamine is a neurotransmitter synthesized in nerve tissue and non-nerve tissues, such as the kidney, and its concentration varies with salt intake and intracellular sodium concentration. It plays a diuretic and natriuretic role by acting on dopamine receptors (DRs) and plays a crucial function in keeping blood pressure in equilibrium [58]. The DR family includes D1-like receptors (D1R, D5R) and D2-like receptors (D2R, D3R, D4R). The D1-like receptor interacts with the Gs protein to activate adenosine cyclase and increase cAMP production, thereby inhibiting the Na+/H+ exchanger and Na+/K+/ATPase pump and increasing urinary sodium excretion. The interaction of the D2-like receptor with the Gi protein has a completely opposite effect to the D1-like receptor action above [59,60]. When the body ingests a high-salt diet, the dopaminergic system in the kidney is activated, and Na+ transport in epithelial cells is reduced. At this time, the RAAS is activated to counteract the reduction in Na+ transport to maintain sodium homeostasis [61]. When the renal dopaminergic system is abnormal, RAAS will also be disrupted, inducing salt-sensitive hypertension; hence, the renal dopaminergic system and RAAS are very important for dopamine to exert blood pressure homeostasis and intracellular sodium balance [60]. In addition, dopamine regulates mitochondrial function, increases autophagy and reduces the generation of reactive oxygen species by the cAMP-PKA signaling pathway and the DR, which also plays a crucial function in the pathogenesis of hypertension [62,63]. Many studies have shown that animals/human hypertension/salt-sensitive hypertension is closely related to the abnormal regulation of dopamine receptors or dopamine production [60,64,65], and abnormal dopamine receptors may be related to increased G protein receptor kinase 4 activity [66,67]. Similarly, disruption of any dopamine receptor in mice causes high blood pressure [58]. According to our study, dopamine levels showed different degrees of increase in each hypertension group, but cAMP in the downstream pathway was down-regulated. The inhibition of this dopamine downstream pathway is likely to be caused by the abnormality of a DR, which reduces renal sodium excretion ability and causes hypertension; it is not clear which DR subtype is involved. In addition, other altered metabolites may also have a function in the development of pediatric hypertension. Another study also applied metabolomics methods and found that hypertensive patients with renal fibrosis, endothelial dysfunction and atherosclerosis also exhibited serine, glycine, methionine and homocysteine metabolism disorders, and these changes had a direct link with blood pressure [68]. 3-methoxytyramine can induce intracellular reactive oxygen species production, resulting in cerebral ischemia injury [69]. Glycine can increase renal blood flow and renal cortex perfusion in SD rats, providing a significant sodium and diuretic mitigation effect on hypertension [70].

Our current research has some limitations. First, age, sex, and the relatively small sample size can skew results. Future research will need to include a larger population and ensure a balanced number of boys and girls. Second, there was limited screening of healthy children providing samples for the control group, and careful selection should avoid other symptoms or diseases interfering with metabolomics results. Third, most of the children with simple and H-type hypertension were overweight or obese. What caused these children to become obese was not explored, and hypertension-related pathways caused by lipid metabolism disorders have not been found. Fourth, it is not clear if genetic mutations, issues with diet or a lack of folic acid or vitamin B12 were present in children with hypertension exhibiting elevated plasma Hcy concentrations. In addition, it is not clear whether the relationship between damage to the renal dopaminergic system and hypertension was caused by gene mutations or other factors. In view of these limitations, we hope to further explore these findings through animal models and by expanding the number of human samples in future studies.

## 5. Conclusions

In summary, we found that lipid metabolism disorders occurred in all children with hypertension, and most of them had central obesity. Hypertensive children with a normal BMI may also have a tendency towards obesity. In addition, elevated plasma Hcy levels may make lipid metabolism disorders more serious and increase insulin resistance, exacerbating the severity of hypertension. For overweight or obese children with high blood pressure, strengthening physical exercise and eating less to maintain a normal BMI may help to maintain a normal blood pressure. For children with H-type hypertension, reducing the concentration of plasma Hcy may provide a better outcome regarding blood pressure, and understanding the specific mechanism needs further research. In summary, our research serves as a foundation for further investigation into the pathophysiological basis of hypertension and the effect of plasma Hcy on young patients’ blood pressure.

## Figures and Tables

**Table 1:** Description of basic information of all children.

Groups	Cases, n	M/F, n	Age ($\bar{\chi }$ ± s, year)	sSBP ($\bar{\chi }$ ± s, mmHg)	sDBP ($\bar{\chi }$ ± s, mmHg)	Hcy [M(Q_1_, Q_3_), μmol/L]	BMI ($\bar{\chi }$ ± s, kg/m^2^)
C	29	13/16	11.31 ± 2.47	107.10 ± 11.23	66.48 ± 7.89	7.72 (3.01,9.10)	20.62 ± 5.04
NBp	38	6/2	12.5 ± 2.33	129.38 ± 9.84 ^***^	79.25 ± 7.85 ^***^	7.57 (7.14,7.92)	20.75 ± 3.32
OBp	8	30/8 ^**^	12.26 ± 2.16	138.76 ± 11.37 ^***^	77.61 ± 8.97 ^***^	8.25 (6.92,9.18)	29.00 ± 3.18 ^***, +++^
NH	37	15/4 ^*^	13.21 ± 1.81 ^*^	136.42 ± 12.62 ^***^	80.21 ± 10.87 ^***^	12.05 (10.89,18.51) ^****, ++, Ϯ Ϯ Ϯ^	21.19 ± 1.87 ^Ϯ Ϯ Ϯ^
OH	19	33/4 ^***^	13.65 ± 1.70 ^***, Ϯ^	140.84 ± 10.53 ^***^	82.27 ± 9.47 ^***^	12.77 (11.34,16.44) ^****, ++, Ϯ Ϯ Ϯ^	29.57 ± 4.03 ^***, +++, ^^^^
χ/F/K	-	17.108	5.801	45.698	13.448	87.782	40.057
*p* value	-	0.002	0.000	0.000	0.000	0.000	0.000

sSBP: mean supine systolic blood pressure; sDBP: mean supine diastolic blood pressure; Hcy: homocysteine. BMI: body mass index; C: control group; NBp: normal body mass index and simple hypertension group; OBp: overweight with simple hypertension group; NH: normal body mass index and H-type hypertension group; OH: overweight with H-type hypertension group. * *p* < 0.05, ** *p* < 0.01, *** *p* < 0.001 and **** *p* < 0.0001 vs. the control group; ^++^ *p* < 0.01, ^+++^ *p* < 0.001 vs. the NBp group; ^Ϯ^ *p* < 0.05 and ^ϮϮϮ^ *p* < 0.001 vs. the OBp group; ^^^^^ *p* < 0.001 vs. the NH group.

**Table 2:** Some significantly different metabolites between hypertension groups and normal control.

Adduct	Description	VIP	FC	*p*-Value	m/z	rt (s)
[M − H]^−^	DHEAS	2.014	2.247	5.321 × 10^-5^	367.15834	30.48445
[M + H − 3H_2_O]^+^	gamma.-muricholic acid	1.322	0.077	3.689 × 10^-18^	355.26306	212.2305
[M − H]^−^	2-hydroxyisocaproic acid	1.793	1.825	3.466 × 10^−5^	131.07144	109.993
[M + H]^+^	Adenosine	3.893	0.712	0.045	268.12684	234.468
(M – H + 2Na)^+^	Acetylcholine	1.317	0.457	8.614 × 10^−6^	190.08222	109.7705
[M + H]^+^	Adrenosterone	3.243	86.837	0.001	301.16932	126.52
[M − H]^−^	Dopamine	2.686	12.250	0.003	152.00228	185.4145
[M + H]^+^	DL-Isoleucine	1.431	0.297	2.219 × 10^−9^	132.1018	167.901
[M − H]^−^	Glycine	2.186	0.439	1.008 × 10^−5^	74.02479	43.7139
(M + H)^+^	ADMA	1.776	1.747	3.930 × 10^−5^	203.15034	598.283
[M + H − H_2_O]^+^	cAMP	2.217	0.290	4.750 × 10^−5^	312.05942	312.74
[M + H − H_2_O]^+^	Monoethyl Phthalate	3.954	1.374	0.031	177.0407	181.5925
[M + H − NH_3_]^+^	3-methoxytyramine	2.418	4.740	0.041	151.06127	77.34585
[M − H − C_7_H_6_O]^−^	Phloretin	1.192	0.613	0.025	167.03638	46.9116

Adduct: adduct ion information of compounds; VIP: variable importance in the projection; FC: fold change; m/z: mass-to-charge ratio; rt (s): the peak time, in seconds; DHEAS: 3-dehydroepiandrosterone sulfate; ADMA: NG, NG-dimethyl-L-arginine; cAMP: adenosine 3′,5′-cyclic monophosphate.

## Data Availability

The data used in this study can be provided by the corresponding authors according to reasonable requirements.

## Supplementary Materials

The following supporting information can be downloaded at: https://www.mdpi.com/article/10.3390/nu15040836/s1, Figure S1: Differences in metabolites between and within groups. Figure S2: Network of compound–reaction–enzyme–gene related to Hcy metabolism. Figure S3: KEGG pathway map of insulin resistance. Figure S4: Pathway diagram related to hypertension.

## References

[1] Joint Committee for Guideline Revision (2019). 2018 Chinese Guidelines for Prevention and Treatment of Hypertension-A report of the Revision Committee of Chinese Guidelines for Prevention and Treatment of Hypertension. J. Geriatr. Cardiol..

[2] Lurbe E., Agabiti-Rosei E., Cruickshank J.K., Dominiczak A., Erdine S., Hirth A., Invitti C., Litwin M., Mancia G., Pall D. (2016). 2016 European Society of Hypertension guidelines for the management of high blood pressure in children and adolescents. J. Hypertens..

[3] Falkner B. (2022). The enigma of primary hypertension in childhood. Front. Cardiovasc. Med..

[4] Song P., Zhang Y., Yu J., Zha M., Zhu Y., Rahimi K., Rudan I. (2019). Global Prevalence of Hypertension in Children: A Systematic Review and Meta-analysis. JAMA Pediatr..

[5] Dong J., Dong H., Yan Y., Cheng H., Zhao X., Mi J. (2022). Prevalence of hypertension and hypertension phenotypes after three visits in Chinese urban children. J. Hypertens..

[6] Gunn H.E., Eberhardt K.R. (2019). Family Dynamics in Sleep Health and Hypertension. Curr. Hypertens. Rep..

[7] Guzman-Limon M., Samuels J. (2019). Pediatric Hypertension: Diagnosis, Evaluation, and Treatment. Pediatr. Clin. N. Am..

[8] Furusho T., Uchida S., Sohara E. (2020). The WNK signaling pathway and salt-sensitive hypertension. Hypertens. Res..

[9] Kho J., Tian X., Wong W.T., Bertin T., Jiang M.M., Chen S., Jin Z., Shchelochkov O.A., Burrage L.C., Reddy A.K. (2018). Argininosuccinate Lyase Deficiency Causes an Endothelial-Dependent Form of Hypertension. Am. J. Hum. Genet..

[10] Ercu M., Markó L., Schächterle C., Tsvetkov D., Cui Y., Maghsodi S., Bartolomaeus T.U., Maass P.G., Zühlke K., Gregersen N. (2020). Phosphodiesterase 3A and Arterial Hypertension. Circulation.

[11] Guo J., Wang Z., Wu J., Liu M., Li M., Sun Y., Huang W., Li Y., Zhang Y., Tang W. (2019). Endothelial SIRT6 Is Vital to Prevent Hypertension and Associated Cardiorenal Injury Through Targeting Nkx3.2-GATA5 Signaling. Circ. Res..

[12] Krivosikova K., Krivosikova Z., Wsolova L., Seeman T., Podracka L. (2022). Hypertension in obese children is associated with vitamin D deficiency and serotonin dysregulation. BMC Pediatr..

[13] Stanek A., Brozyna-Tkaczyk K., Myslinski W. (2021). The Role of Obesity-Induced Perivascular Adipose Tissue (PVAT) Dysfunction in Vascular Homeostasis. Nutrients.

[14] Zhang R.M., McNerney K.P., Riek A.E., Bernal-Mizrachi C. (2021). Immunity and Hypertension. Acta Physiol..

[15] Zhang L., Li Z., Xing C., Gao N., Xu R. (2021). Folate Reverses NF-kappaB p65/Rela/IL-6 Level Induced by Hyperhomocysteinemia in Spontaneously Hypertensive Rats. Front. Pharmacol..

[16] Boachie J., Adaikalakoteswari A., Samavat J., Saravanan P. (2020). Low Vitamin B12 and Lipid Metabolism: Evidence from Pre-Clinical and Clinical Studies. Nutrients.

[17] Nicholson J.K., Lindon J.C., Holmes E. (1999). ‘Metabonomics’: Understanding the metabolic responses of living systems to pathophysiological stimuli via multivariate statistical analysis of biological NMR spectroscopic data. Xenobiotica.

[18] Liu H., Garrett T.J., Su Z., Khoo C., Gu L. (2017). UHPLC-Q-Orbitrap-HRMS-based global metabolomics reveal metabolome modifications in plasma of young women after cranberry juice consumption. J. Nutr. Biochem..

[19] Griffin J.L. (2006). The Cinderella story of metabolic profiling: Does metabolomics get to go to the functional genomics ball?. Philos. Trans. R. Soc. B. Biol. Sci..

[20] Du Y., Hou L., Chu C., Jin Y., Sun W., Zhang R. (2019). Characterization of serum metabolites as biomarkers of carbon black nanoparticles-induced subchronic toxicity in rats by hybrid triple quadrupole time-of-flight mass spectrometry with non-targeted metabolomics strategy. Toxicology.

[21] Mu X., Ji C., Wang Q., Liu K., Hao X., Zhang G., Shi X., Zhang Y., Gonzalez F.J., Wang Q. (2019). Non-targeted metabolomics reveals diagnostic biomarker in the tongue coating of patients with chronic gastritis. J. Pharm. Biomed. Anal..

[22] Averina M., Brox J., Huber S., Furberg A.S. (2021). Exposure to perfluoroalkyl substances (PFAS) and dyslipidemia, hypertension and obesity in adolescents. The Fit Futures study. Environ. Res..

[23] Sun J., Zhao M., Yang L., Liu X., Pacifico L., Chiesa C., Xi B. (2021). Identification of Potential Metabolic Markers of Hypertension in Chinese Children. Int. J. Hypertens..

[24] Wang L., Hou E., Wang L., Wang Y., Yang L., Zheng X., Xie G., Sun Q., Liang M., Tian Z. (2015). Reconstruction and analysis of correlation networks based on GC-MS metabolomics data for young hypertensive men. Anal. Chim. Acta.

[25] Akyurek N., Atabek M.E., Eklioglu B.S., Alp H. (2015). Is there a relationship between cardiovascular risk factors and dehydroepiandrosterone sulfate levels in childhood obesity?. J. Pediatr. Endocrinol. Metab..

[26] Topsakal S., Akin F., Yerlikaya E., Erurker T., Dogu H. (2014). Dehydroepiandrosterone sulfate levels in Turkish obese patients. Eat. Weight. Disord..

[27] Santos-Silva R., Fontoura M., Guimaraes J.T., Barros H., Santos A.C. (2022). Association of dehydroepiandrosterone sulfate, birth size, adiposity and cardiometabolic risk factors in 7-year-old children. Pediatr. Res..

[28] Hirokawa K., Ohira T., Nagayoshi M., Kajiura M., Imano H., Kitamura A., Kiyama M., Okada T., Iso H. (2016). Dehydroepiandrosterone-sulfate is associated with cardiovascular reactivity to stress in women. Psychoneuroendocrinology.

[29] Zhang M., Zhang M., Wang W., Chen H., Wang X., Zhao K., Li Z., Xu J., Fei T. (2022). Dehydroepiandrosterone inhibits vascular proliferation and inflammation by modulating the miR-486a-3p/NLRP3 axis. Am. J. Transl. Res..

[30] Mannella P., Simoncini T., Caretto M., Genazzani A.R. (2018). Dehydroepiandrosterone and Cardiovascular Disease. Vitam. Horm..

[31] Sakko M., Rautemaa-Richardson R., Sakko S., Richardson M., Sorsa T. (2021). Antibacterial Activity of 2-Hydroxyisocaproic Acid (HICA) against Obligate Anaerobic Bacterial Species Associated with Periodontal Disease. Microbiol. Insights.

[32] Mindikoglu A.L., Opekun A.R., Putluri N., Devaraj S., Sheikh-Hamad D., Vierling J.M., Goss J.A., Rana A., Sood G.K., Jalal P.K. (2018). Unique metabolomic signature associated with hepatorenal dysfunction and mortality in cirrhosis. Transl. Res..

[33] Dhuper S., Buddhe S., Patel S. (2013). Managing cardiovascular risk in overweight children and adolescents. Paediatr. Drugs.

[34] Binka E., Brady T.M. (2019). Real-World Strategies to Treat Hypertension Associated with Pediatric Obesity. Curr. Hypertens. Rep..

[35] Lu G., Meier K.E., Jaffa A.A., Rosenzweig S., Egan B.M. (1998). Oleic acid and angiotensin II induce a synergistic mitogenic response in vascular smooth muscle cells. Hypertension. Hypertension.

[36] Yun M.R., Lee J.Y., Park H.S., Heo H.J., Park J.Y., Bae S.S., Hong K.W., Sung S.M., Kim C.D. (2006). Oleic acid enhances vascular smooth muscle cell proliferation via phosphatidylinositol 3-kinase/Akt signaling pathway. Pharmacol. Res..

[37] Gremmels H., Bevers L.M., Fledderus J.O., Braam B., van Zonneveld A.J., Verhaar M.C., Joles J.A. (2015). Oleic acid increases mitochondrial reactive oxygen species production and decreases endothelial nitric oxide synthase activity in cultured endothelial cells. Eur. J. Pharmacol..

[38] Zhang H.Y., Reddy S., Kotchen T.A. (1999). A high sucrose, high linoleic acid diet potentiates hypertension in the Dahl salt sensitive rat. Am. J. Hypertens..

[39] Mahmmoud Y.A., Christensen S.B. (2011). Oleic and linoleic acids are active principles in Nigella sativa and stabilize an E(2)P conformation of the Na,K-ATPase. Fatty acids differentially regulate cardiac glycoside interaction with the pump. Biochim. Biophys. Acta.

[40] Jovanovich A., Isakova T., Block G., Stubbs J., Smits G., Chonchol M., Miyazaki M. (2018). Deoxycholic Acid, a Metabolite of Circulating Bile Acids, and Coronary Artery Vascular Calcification in CKD. Am. J. Kidney Dis..

[41] Kimber C., Zhang S., Johnson C., West R.E., Prokopienko A.J., Mahnken J.D., Yu A.S., Hoofnagle A.N., Ir D., Robertson C.E. (2020). Randomized, Placebo-Controlled Trial of Rifaximin Therapy for Lowering Gut-Derived Cardiovascular Toxins and Inflammation in CKD. Kidney360.

[42] Wierema T.K., Houben A.J., Kroon A., Postma C.T., Koster D., van Engelshoven J.M., Smits P., de Leeuw P.W. (2005). Mechanisms of adenosine-induced renal vasodilatation in hypertensive patients. J. Hypertens.

[43] Zamora A.N., Jansen E.C., Tamayo-Ortiz M., Goodrich J.M., Sanchez B.N., Watkins D.J., Tamayo-Orozco J.A., Tellez-Rojo M.M., Mercado-Garcia A., Baylin A. (2021). Exposure to Phenols, Phthalates, and Parabens and Development of Metabolic Syndrome Among Mexican Women in Midlife. Front. Public Health.

[44] Soomro M.H., Maesano C.N., Heude B., Bornehag C.-G., Annesi-Maesano I. (2021). The association between maternal urinary phthalate metabolites concentrations and pregnancy induced hypertension: Results from the EDEN Mother-Child Cohort. J. Gynecol. Obstet. Hum. Reprod..

[45] Sawh M.C., Wallace M., Shapiro E., Goyal N.P., Newton K.P., Yu E.L., Bross C., Durelle J., Knott C., Gangoiti J.A. (2021). Dairy Fat Intake, Plasma Pentadecanoic Acid, and Plasma Iso-heptadecanoic Acid Are Inversely Associated with Liver Fat in Children. J. Pediatr. Gastroenterol. Nutr..

[46] Liang J., Zhou Q., Amakye W.K., Su Y., Zhang Z. (2018). Biomarkers of dairy fat intake and risk of cardiovascular disease: A systematic review and meta analysis of prospective studies. Crit. Rev. Food Sci. Nutr..

[47] Ying Y., Jiang C., Zhang M., Jin J., Ge S., Wang X. (2019). Phloretin protects against cardiac damage and remodeling via restoring SIRT1 and anti-inflammatory effects in the streptozotocin-induced diabetic mouse model. Aging.

[48] Hsu C.-N., Tain Y.-L. (2021). Asymmetric Dimethylarginine (ADMA) in Pediatric Renal Diseases: From Pathophysiological Phenomenon to Clinical Biomarker and Beyond. Children.

[49] Lopez V., Uribe E., Moraga F.A. (2021). Activation of arginase II by asymmetric dimethylarginine and homocysteine in hypertensive rats induced by hypoxia: A new model of nitric oxide synthesis regulation in hypertensive processes?. Hypertens. Res..

[50] Lopez V., Moraga F.A., Llanos A.J., Ebensperger G., Taborda M.I., Uribe E. (2018). Plasmatic Concentrations of ADMA and Homocystein in Llama (Lama glama) and Regulation of Arginase Type II: An Animal Resistent to the Development of Pulmonary Hypertension Induced by Hypoxia. Front. Physiol..

[51] Chien S.J., Lin I.C., Hsu C.N., Lo M.H., Tain Y.L. (2015). Homocysteine and Arginine-to-Asymmetric Dimethylarginine Ratio Associated With Blood Pressure Abnormalities in Children With Early Chronic Kidney Disease. Circ. J..

[52] Tousoulis D., Bouras G., Antoniades C., Marinou K., Papageorgiou N., Miliou A., Hatzis G., Stefanadi E., Tsioufis C., Stefanadis C. (2011). Methionine-induced homocysteinemia impairs endothelial function in hypertensives: The role of asymmetrical dimethylarginine and antioxidant vitamins. Am. J. Hypertens..

[53] Dovinova I., Hrabarova E., Jansen E., Kvandova M., Majzunova M., Berenyiova A., Barancik M. (2018). ADMA, homocysteine and redox status improvement affected by 7-nitroindazole in spontaneously hypertensive rats. Biomed. Pharmacother..

[54] Antoniades C., Shirodaria C., Leeson P., Antonopoulos A., Warrick N., Van-Assche T., Cunnington C., Tousoulis D., Pillai R., Ratnatunga C. (2009). Association of plasma asymmetrical dimethylarginine (ADMA) with elevated vascular superoxide production and endothelial nitric oxide synthase uncoupling: Implications for endothelial function in human atherosclerosis. Eur. Heart J..

[55] Wierzbicki A.S. (2007). Homocysteine and cardiovascular disease: A review of the evidence. Diab. Vasc. Dis. Res..

[56] Shi L., Liu X.-Y., Huang Z.-G., Ma Z.-Y., Xi Y., Wang L.-Y., Sun N.-L. (2019). Endogenous hydrogen sulfide and ERK1/2-STAT3 signaling pathway may participate in the association between homocysteine and hypertension. J. Geriatr. Cardiol..

[57] Zhang Y., Wang G., Liu J., Xu Y. (2018). Impact of hyperhomocysteinemia on insulin resistance in patients with H-type hypertension. Clin. Exp. Hypertens..

[58] Yang J., Villar V.A.M., Jose P.A., Zeng C. (2021). Renal Dopamine Receptors and Oxidative Stress: Role in Hypertension. Antioxid. Redox Signal..

[59] Contreras F., Fouillioux C., Bolivar A., Simonovis N., Hernandez-Hernandez R., Armas-Hernandez M.J., Velasco M. (2002). Dopamine, hypertension and obesity. J. Hum. Hypertens..

[60] Martinez V.J., Asico L.D., Jose P.A., Tiu A.C. (2020). Lipid Rafts and Dopamine Receptor Signaling. Int. J. Mol. Sci..

[61] Natarajan A.R., Eisner G.M., Armando I., Browning S., Pezzullo J.C., Rhee L., Dajani M., Carey R.M., Jose P.A. (2016). The Renin-Angiotensin and Renal Dopaminergic Systems Interact in Normotensive Humans. J. Am. Soc. Nephrol..

[62] Lee H., Jiang X., Perwaiz I., Yu P., Wang J., Wang Y., Huttemann M., Felder R.A., Sibley D.R., Polster B.M. (2021). Dopamine D(5) receptor-mediated decreases in mitochondrial reactive oxygen species production are cAMP and autophagy dependent. Hypertens. Res..

[63] Qaddumi W.N., Jose P.A. (2021). The Role of the Renal Dopaminergic System and Oxidative Stress in the Pathogenesis of Hypertension. Biomedicines.

[64] Zeng C., Xia T., Zheng S., Liang L., Chen Y. (2022). Synergistic Effect of Uroguanylin and D(1) Dopamine Receptors on Sodium Excretion in Hypertension. J. Am. Heart Assoc..

[65] Gildea J.J., Xu P., Kemp B.A., Carey R.M., Jose P.A., Felder R.A. (2019). The Dopamine D. 1 Receptor and Angiotensin II Type-2 Receptor are Required for Inhibition of Sodium Transport Through a Protein Phosphatase 2A Pathway. Hypertension.

[66] Harris R.C. (2012). Abnormalities in renal dopamine signaling and hypertension: The role of GRK4. Curr. Opin. Nephrol. Hypertens..

[67] Harris R.C., Zhang M.Z. (2012). Dopamine, the kidney, and hypertension. Curr. Hypertens. Rep..

[68] Ovrehus M.A., Bruheim P., Ju W., Zelnick L.R., Langlo K.A., Sharma K., de Boer I.H., Hallan S.I. (2019). Gene Expression Studies and Targeted Metabolomics Reveal Disturbed Serine, Methionine, and Tyrosine Metabolism in Early Hypertensive Nephrosclerosis. Kidney Int. Rep..

[69] Khan A., Shin M.S., Jee S.H., Park Y.H. (2020). Global metabolomics analysis of serum from humans at risk of thrombotic stroke. Analyst.

[70] Badzynska B., Zakrocka I., Sadowski J., Turski W.A., Kompanowska-Jezierska E. (2014). Effects of systemic administration of kynurenic acid and glycine on renal haemodynamics and excretion in normotensive and spontaneously hypertensive rats. Eur. J. Pharmacol..

