# Supplementary material for: Untargeted Metabolomics Analysis Using UHPLC-Q-TOF/MS Reveals Metabolic Changes Associated with Hypertension in Children
Source: Nutrients. 2023 Feb 6;15(4):836. doi: 10.3390/nu15040836 (PMC9964282; doi:10.3390/nu15040836)
Supplement: Supplementary file 1 [file nutrients-15-00836-s001.zip › nutrients-2182690-supplementary.pdf]

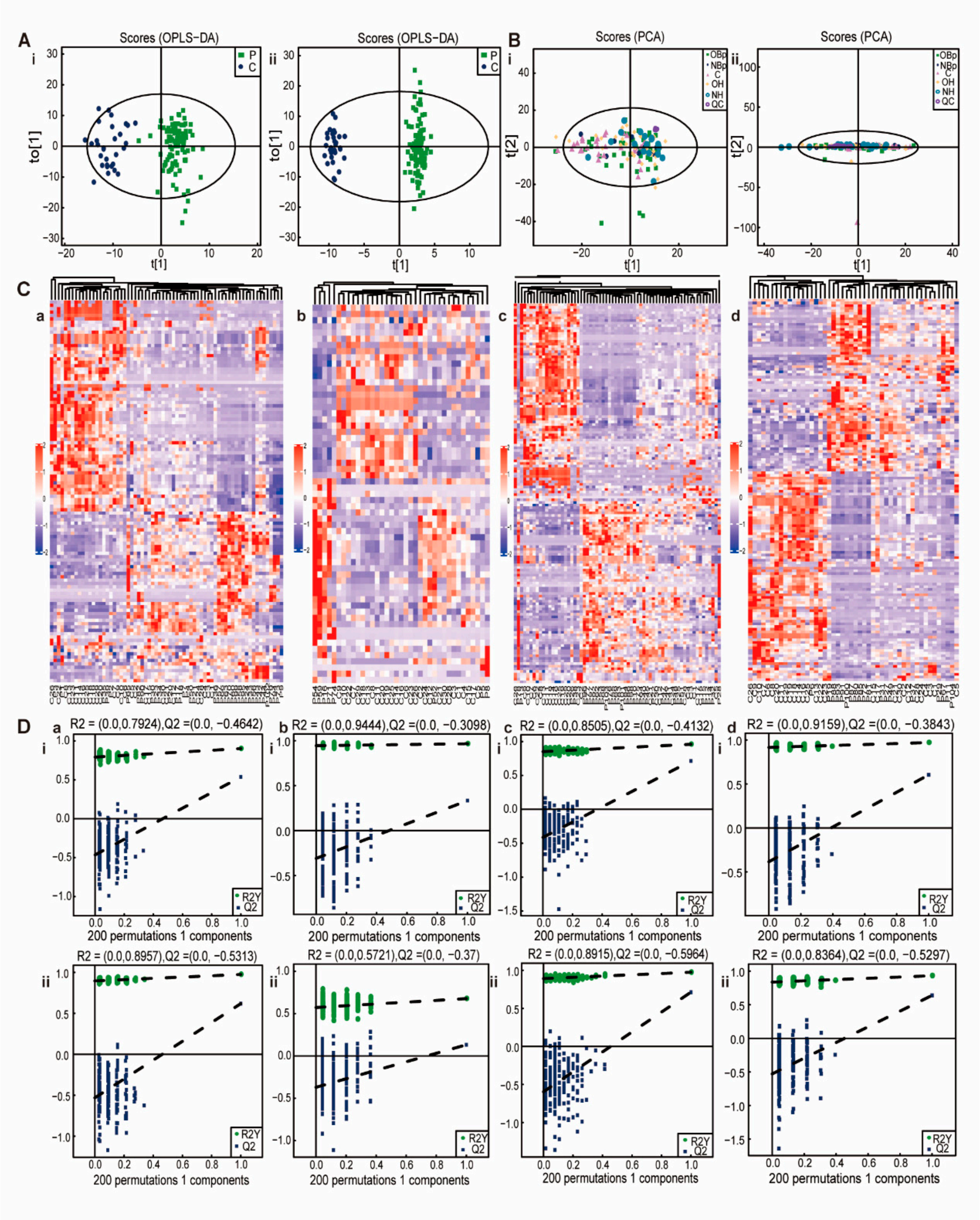

Figure S1. Differences in metabolites between and within groups.

A (i, ii) OPLS-DA score plot of the positive ion mode and negative ion mode. The t[1] represents the samples' score on the first principal component to distinguish between groups, and the to[1] represents the samples' score on the second principal component to reflect the intra-group variation. The ellipse represents 95% confidence interval. Points of the same color represent biological replicates within a group, and the distribution of points reflects the degree of difference between and within groups. P: hypertension group; C: control group.

B (i, ii) PCA score plot of the positive ion mode and negative ion mode. The ellipse represents 95% confidence interval. The

t[1] represents the samples' score on the first principal component, and the t[2] represents the samples' score on the second principal component. Green squares represent each biological repetition in the OBp group. Dark blue circles represent each biological repetition in the NBp group. Purple triangles represent each biological repetition in the control group. Yellow points represent each biological repetition in the OH group. Light blue circles represent each biological repetition in the NH group and purple circles represent QC samples.

C Heatmap of positive ion mode. Each column in the x-axis represents a set of samples. The metabolites with similar expression patterns are clustered in the same cluster on the left. Red represents the significant up-regulation of the metabolite and purple represents the significant down-regulation of the metabolite. Color depth represents the degree of up-regulation and down-regulation. a: OBp vs. C; b: NBp vs. C; c: OH vs. C; d: NH vs. C.

D The permutation test of OPLS-DA models. In the figure, i and ii represent positive ion mode and negative ion mode. The abscissa represents the replacement retention, that is, the proportion consistent with the original model Y variable order, and the ordinate represents the values of R2 and Q2. The green point represents R2, the blue point represents Q2, and the two dashed lines represent the regression lines of R2 and Q2, respectively. R2 and Q2 in the upper right corner indicate that the replacement retention is equal to 1, that is, the R2 and Q2 values of the original model. As the permutation retention decreases, both R2 and Q2 of the stochastic model decrease. a: OBp vs. C; b: NBp vs. C; c: OH vs. C; d: NH vs. C.

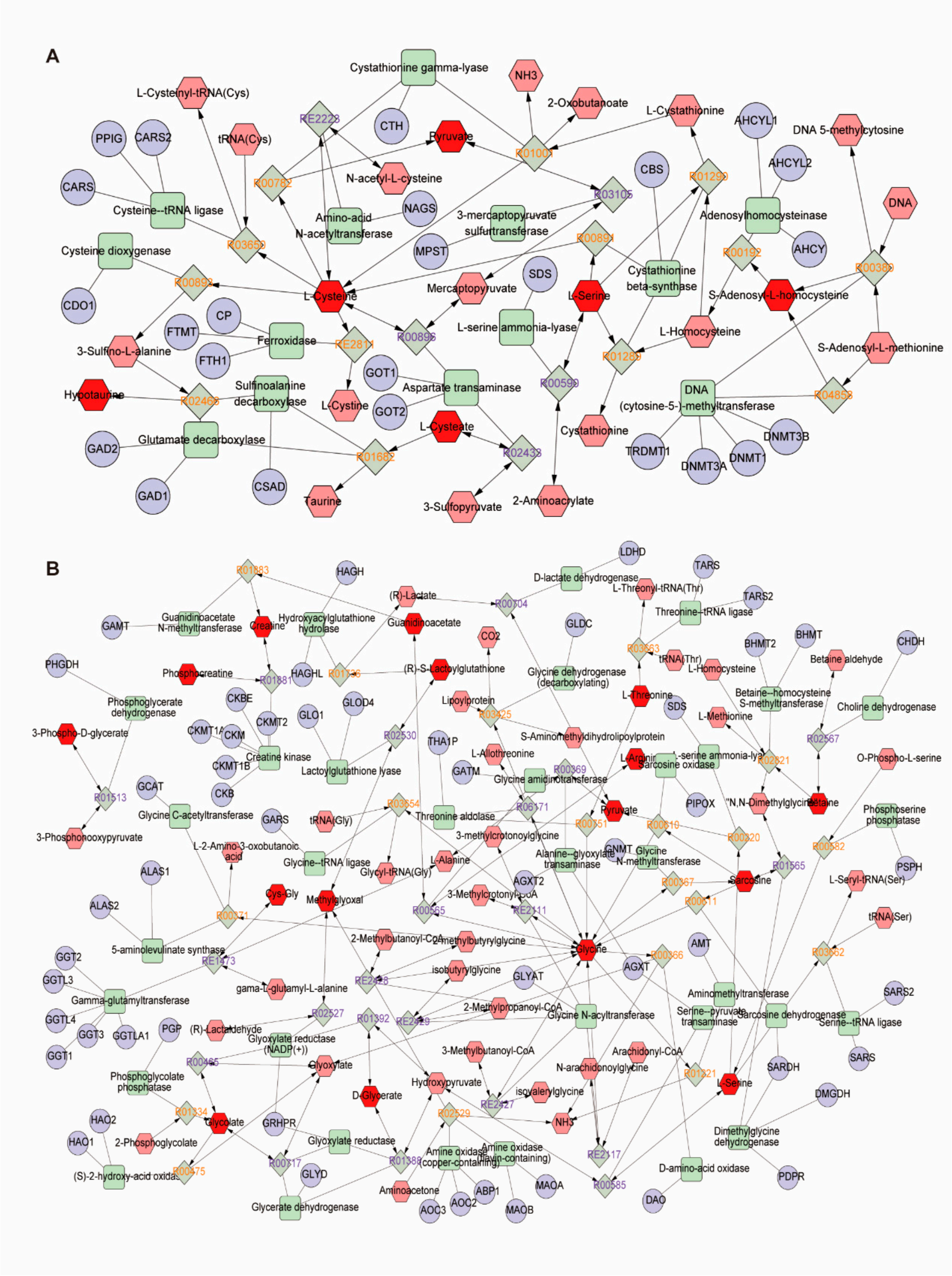

Figure S2. Network of compound-reaction-enzyme-gene related to Hcy metabolism.

The hexagons represent compounds. Circles represent genes. Squares represent the enzymes. The diamonds indicate the reaction between multiple compounds and genes. The red hexagons indicate the up-regulated or down-regulated differential metabolites screened in this experiment. **A**: Methionine cysteine metabolism. **B**: Glycine, serine, alanine, threonine metabolism.

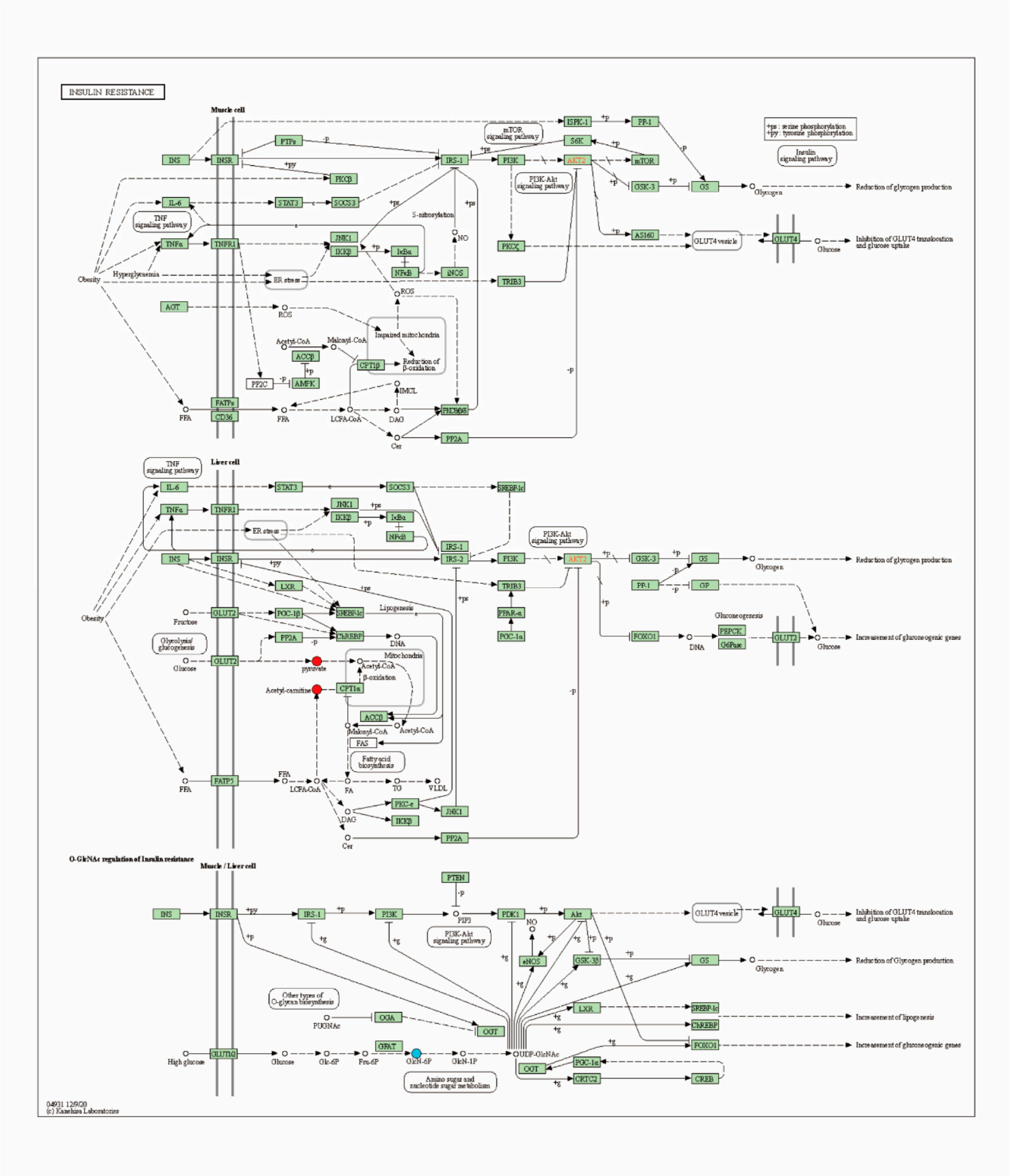

Figure S3. KEGG pathway map of insulin resistance.

Blue circles in the metabolic pathway map indicate down-regulation of metabolites, while red indicates up-regulation. The light green boxes are species-specific proteins.

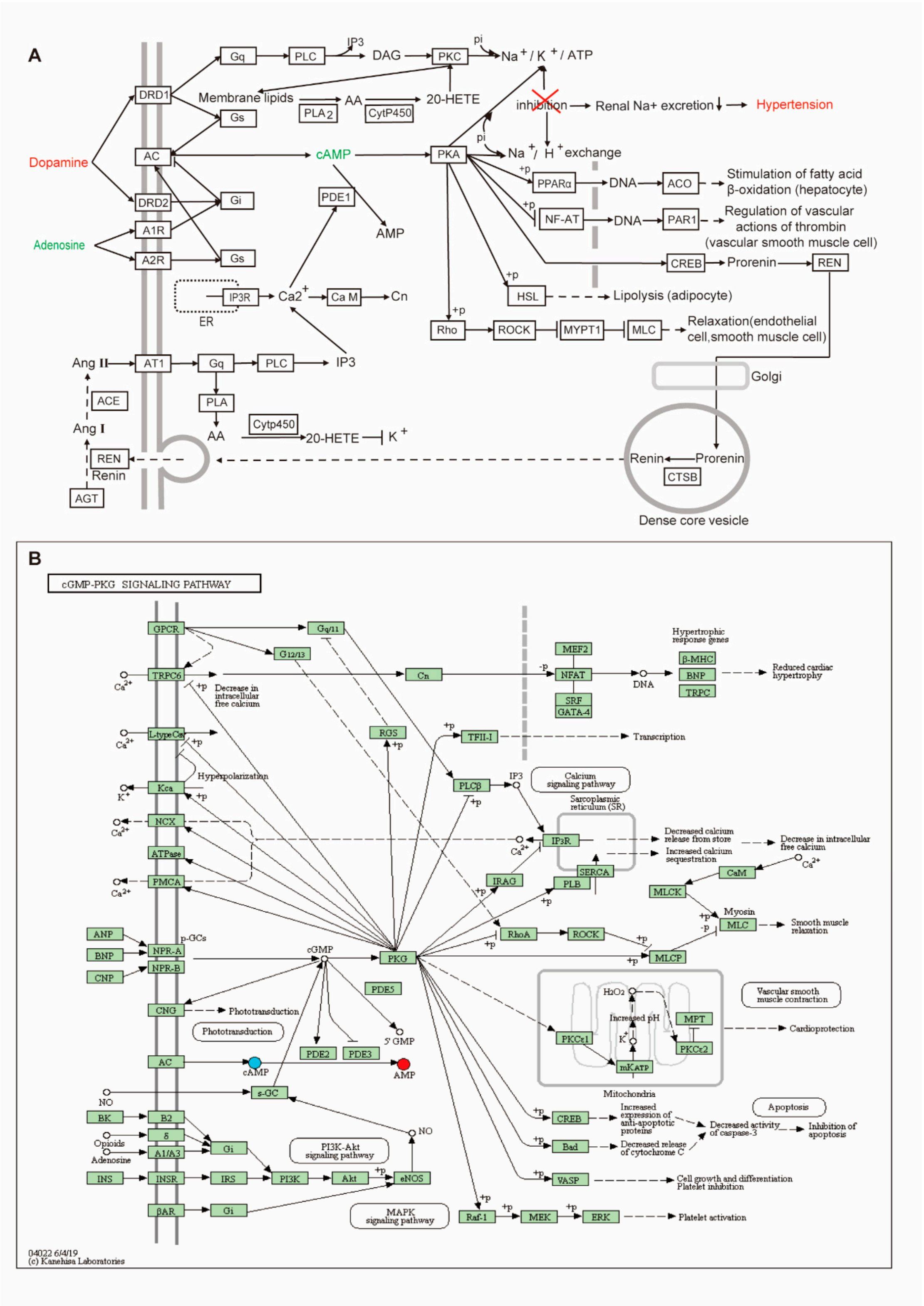

Figure S4. Pathway diagram related to hypertension.

A Dopamine and Adenosine signaling pathway diagram. Enzymes acting on the pathway are shown in the boxes. Red represents up-regulated metabolites or diseases, and green represents down-regulated metabolites.

B KEGG pathway of cGMP-PKG signaling pathway.The blue circles in the metabolic pathway map represent downregulation of metabolites. The light green boxes are species-specific proteins.
